# Functional characterization of *ABCB4* mutations found in progressive familial intrahepatic cholestasis type 3

**DOI:** 10.1038/srep26872

**Published:** 2016-06-03

**Authors:** Hyo Jin Park, Tae Hee Kim, So Won Kim, Shin Hye Noh, Kyeong Jee Cho, Choe Choi, Eun Young Kwon, Yang Ji Choi, Heon Yung Gee, Ji Ha Choi

**Affiliations:** 1 Department of Pharmacology, Tissue Injury Defense Research Center, School of Medicine, Ewha Womans University, Seoul, Korea; 2 Department of Pharmacology and the Institute for Clinical and Translational Research, Catholic Kwandong University College of Medicine, Gangneung, Korea; 3 Department of Pharmacology, Brain Korea 21 PLUS Project for Medical Sciences, Yonsei University College of Medicine, Seoul, Korea

## Abstract

Multidrug resistance 3 (MDR3), encoded by the ATP-binding cassette, subfamily B, member 4 gene (*ABCB4*), localizes to the canalicular membrane of hepatocytes and translocates phosphatidylcholine from the inner leaflet to the outer leaflet of the canalicular membrane. Progressive familial intrahepatic cholestasis type 3 (PFIC3) is a rare hepatic disease caused by genetic mutations of *ABCB4*. In this study, we characterized 8 *ABCB4* mutations found in PFIC3 patients, using *in vitro* molecular assays. First, we examined the transport activity of each mutant by measuring its ATPase activity using paclitaxel or phosphatidylcholine. Then, the pathogenic mechanisms by which these mutations affect MDR3 were examined through immunoblotting, cell surface biotinylation, and immunofluorescence. As a result, three *ABCB4* mutants showed significantly reduced transport activity. Among these mutants, one mutation A364V, located in intracellular domains, markedly decreased MDR3 expression on the plasma membrane, while the others did not affect the expression. The expression of MDR3 on the plasma membrane and transport activity of A364V was rescued by a pharmacological chaperone, cyclosporin A. Our study provides the molecular mechanisms of *ABCB4* mutations and may contribute to the understanding of PFIC3 pathogenesis and the development of a mutation-specific targeted treatment for PFIC3.

The liver is the one of the major organs responsible for the elimination of drugs and other xenobiotics from the body^1^. The ATP-binding cassette, subfamily B, member 4 gene (*ABCB4*), codes for human multidrug resistance 3 (MDR3)^2^. MDR3 is mainly expressed on the canalicular membrane of hepatocytes and plays an important role in the protection of the liver through translocation of phosphatidylcholine from the inner leaflet to the outer leaflet of the canalicular membrane^2^,^3^. Progressive familial intrahepatic cholestasis type 3 (PFIC3) is caused by genetic mutations in *ABCB4*^4^. PFIC denotes a group of rare hepatic diseases, and onset varies from the neonatal period to early adulthood, depending on the specific type of disease. PFIC patients are usually candidates for liver transplantation before adulthood, due to complications such as hepatic failure, liver cirrhosis, and hepatocellular carcinoma^5^. Recently, new approaches for the treatment of hepatic diseases resulting from canalicular transport defects, as occurs in PFIC, have been suggested, including inducing an increase in the expression of functional protein by using synthetic farnesoid X receptor ligands or pharmacological chaperones^6^.

To date, several studies have been conducted to identify and functionally characterize the *ABCB4* mutations found in PFIC3 patients, however, they do not include all the identified mutations^7^,^8^,^9^,^10^,^11^,^12^. A missense mutation found in PFIC3 patients, I541F, was shown to decrease transport activity through reduction of membrane MDR3 expression, which could be functionally rescued by low temperature or cyclosporin A^8^,^9^. Some genetic variations of *ABCB4* are associated with other hepatobiliary diseases such as obstetric cholestasis, cholelithiasis, drug-induced liver injury, and primary biliary cirrhosis^13^,^14^,^15^,^16^,^17^,^18^. Recently, we performed functional characterization of the *ABCB4* promoter variants and found that two common *ABCB4* promoter haplotypes led to significantly decreased promoter activity. In addition, we identified nuclear factor-Y (NF-Y) as a possible transcriptional factor involved in the regulation of *ABCB4* transcription^19^.

Previously, *ABCB4* mutations were newly identified through direct sequencing, using genomic DNA samples from 68 PFIC3 patients^7^. The authors found 29 mutations in the coding region, including 23 missense, four nonsense, and two short insertion mutations. To evaluate the effect of these mutations on protein function, they mapped each variant on the predicted tertiary structure of MDR3, and found that 10 out of 29 mutations were located on transmembrane domains (TMs). In particular, TM 7, which may be required for the translocation of phosphatidylcholine, was most commonly affected. However, these studies did not perform a functional characterization of mutations.

In the present study, we selected 8 missense mutations of *ABCB4* that were first reported by Degiorgio *et al.*^7^ and investigated the effect of each mutant on MDR3 transport activity or expression by using a range of *in vitro* assays such as a membrane vesicular adenosine triphosphatase (ATPase) assay, immunoblotting, surface biotinylation assay, and immunofluorescence. Our study provides the molecular pathogenic mechanisms of *ABCB4* mutations, and our findings may contribute to the development of a new drug for the treatment of PFIC3 or other MDR3-deficiency related diseases.

## Results

### *ABCB4* mutations examined in this study

We selected 8 missense mutations that were first reported by Degiorgio *et al.*^7^. Among the 29 *ABCB4* mutations identified by Degiorgio *et al.*^7^, we excluded nonsense mutations and mutations that were also found in other MDR3-deficient phenotypes or those that affect amino acid residues, which are conserved in other ABC transporters, in particular, the MDR1 transporter, as these had already been investigated^20^,^21^,^22^. The 8 missense *ABCB4* mutations examined in this study are listed in Table 1.

### *ABCB4* mutations affect transporter activity

**Figure 1:**
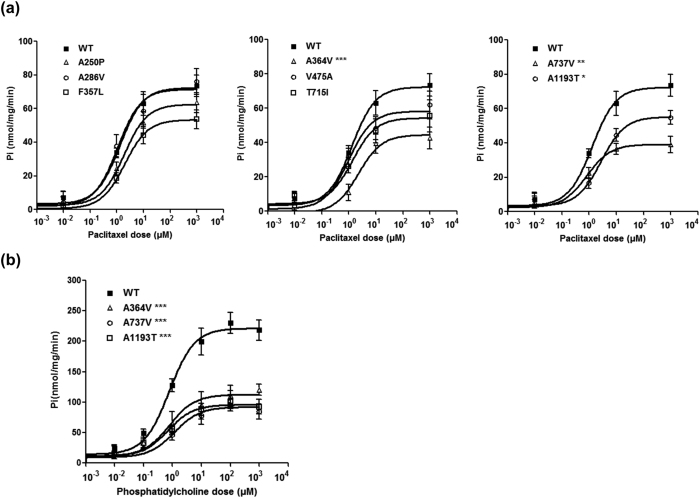
Effect of *ABCB4* mutants on transport activity. Inside-out membrane vesicles were prepared after transfection of *ABCB4* wild type or mutant-bearing plasmids into HEK-293T cells, and the ATPase activity induced by either paclitaxel (**a**) or phosphatidylcholine (**b**) was measured. The X-axis represents paclitaxel or phosphatidylcholine concentration. The Y-axis represents the amount of inorganic phosphate that was produced by the ATPase activity of MDR3. Data shown represent mean ± SD from five independent experiments and analyzed by one-way analysis of variance followed by Dunnett’s two-tailed test. ^*^P < 0.05, ^**^P < 0.01, ^***^P < 0.001 vs. wild type (WT).

To characterize the functional effects of the *ABCB4* mutations, we examined the transport activity of each mutant by measuring the ATPase activity using paclitaxel or phosphatidylcholine, which are known MDR3 substrates^23^,^24^ (Fig. 1). To exclude ATPase activity by other endogenous ABC transporters, such as MDR1, values for transport activity were obtained by subtracting the uptake in empty-vector-transfected cells from that in cells transfected with *ABCB4* wild type or mutant-bearing vectors, at each paclitaxel or phosphatidylcholine concentration. Inhibition of transport by verapamil, a known inhibitor of MDR3^23^, confirmed that the transport was MDR3-mediated (Supplementary Fig. S1). As a result, the paclitaxel-induced ATPase activity was significantly reduced in membrane vesicles, which expressed three *ABCB4* mutations: A364V, A737V, and A1193T (Fig. 1a). Table 2 shows the paclitaxel V_max_ and K_m_ values for the *ABCB4* wild type or mutants. We observed that the average value of V_max_/K_m_ for three *ABCB4* mutants was significantly reduced compared to that of the *ABCB4* wild type. This resulted from a reduced V_max_. MDR3 shows phosphatidylcholine-induced ATPase activity^25^; therefore, we additionally performed the ATPase assay using phosphatidylcholine, for the three mutants: A364V, A737V, and A1193T. We observed a significant decrease in their ATPase activities and this is consistent with the results obtained using paclitaxel (Fig. 1b).

### The effect of mutations on the expression level of MDR3

**Figure 2:**
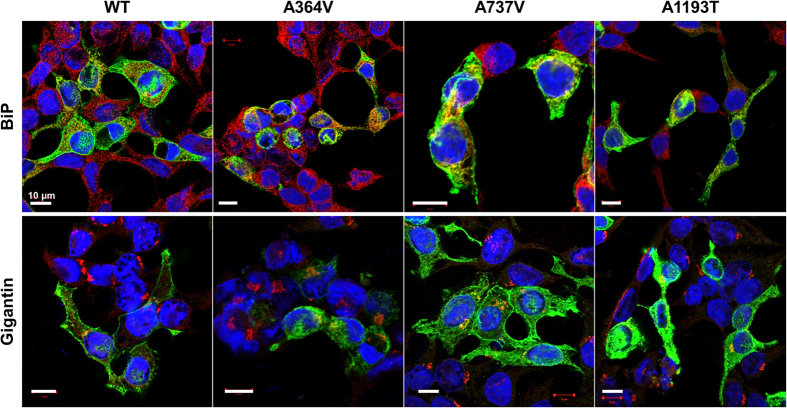
Effect of *ABCB4* mutants on subcellular localization. HEK-293T cells were transfected with *ABCB4* wild type or mutant plasmids. Cells were fixed and permeabilized with methanol, and immunostained with anti-MDR3, and an endoplasmic reticulum maker BiP or a Golgi marker Gigantin antibodies. Scale bars, 10 μm.

**Figure 3:**
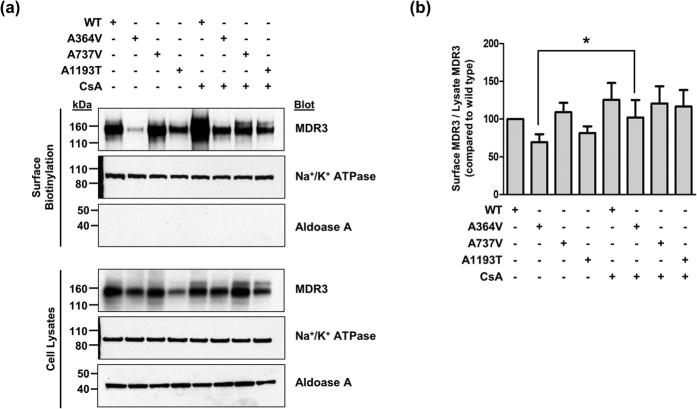
Effect of *ABCB4* mutants and cyclosporin A (CsA) on the surface expression of MDR3. (**a**) HEK-293T cells transfected with *ABCB4* wild type or mutant plasmids, and surface biotinylated. CsA was treated at 10 μM for 24 hours. Absence of the cytosolic protein aldolase A in the biotinylated fraction confirms cell surface protein-specific labeling in each experiment. (**b**) Quantification of surface expression of MDR3. Bar graphs represent band density of surface biotinylated MDR3 compared to the wild type *ABCB4* and error bars indicate the SD for three independent experiments. ^*^P < 0.05, Student’s *t* test.

**Figure 4:**
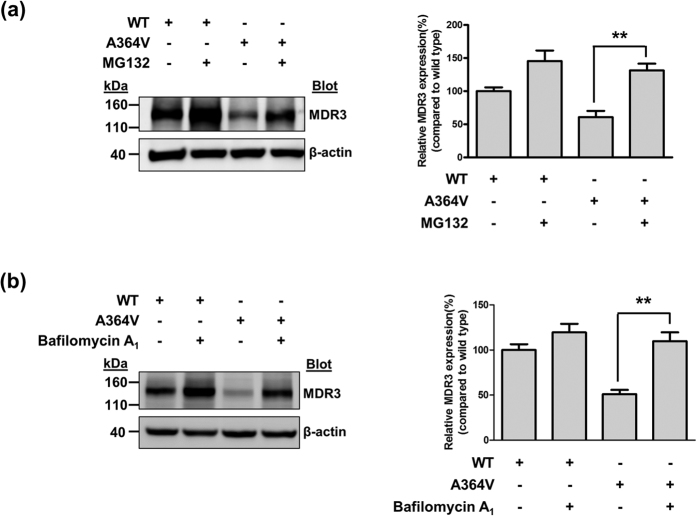
Effect of MG132 or bafilomycin A_1_ on MDR3 expression. MDR3 expression was investigated after transfection with *ABCB4* wild type or A364V mutant plasmids. Immunoblotting was performed after treatment with MG132 (**a**) or bafilomycin A_1_ (**b**). Data shown represent mean ± SD from three independent experiments and analyzed by one-way analysis of variance followed by Dunnett’s two-tailed test. ^**^P < 0.01 vs. expression of A364V without MG132 or bafilomycin A_1_ treatment.

To examine the mechanisms through which *ABCB4* mutations alter the transport activity, we investigated MDR3 expression levels of the mutants on the plasma membrane by immunofluorescence and cell surface biotinylation. Upon overexpression of wild type and mutant *ABCB4* in HEK-293T cells, among the three *ABCB4* mutants that showed decreased transport activities, A737V and A1193T was mainly expressed on the plasma membrane like *ABCB4* wild type (Fig. 2). However, we observed that the MDR3 expression of A364V on the plasma membrane was decreased and a large of fraction of MDR3 was present in the endoplasmic reticulum or Golgi (Fig. 2). In addition, cell surface biotinylation demonstrated that A364V mutant had significantly decreased MDR3 expression on the plasma membrane by 30% as compared to that in the wild type vector transfected cells (Fig. 3). The surface expression level of A737V was comparable with that of the wild type, and that of A1193T seemed to decrease, but it was not statistically significant (Fig. 3), even though their transport activities were decreased. Based on these results, we suspected that A364V mutant had folding or trafficking defects. Proteins with trafficking defects usually undergo degradation by proteasome or lysosome, we examined this for A364V. We observed that MDR3 expression of A364V was significantly increased by 116% and 115%, after treatment with an inhibitor of proteasomal proteolysis, MG132 (Fig. 4a), and an inhibitor of lysosomal degradation, bafilomycin A_1_, respectively (Fig. 4b). While, MDR3 expression of the wild type vector transfected cells was not as much as that for A364V, expression was increased by 46% and 18% after treatment with MG132 and bafilomycin A_1_, respectively (Fig. 4). These data suggest that A364V is vulnerable to intracellular degradation, and the decreased MDR3 expression of this mutant might be a result of proteasomal or lysosomal degradation. For the five mutations that showed similar transport activities as compared to the wild type, their MDR3 expression levels were comparable with those of the wild type (Supplementary Fig. S2).

### The effect of cyclosporin A on the expression and function of a trafficking-defective mutant

**Figure 5:**
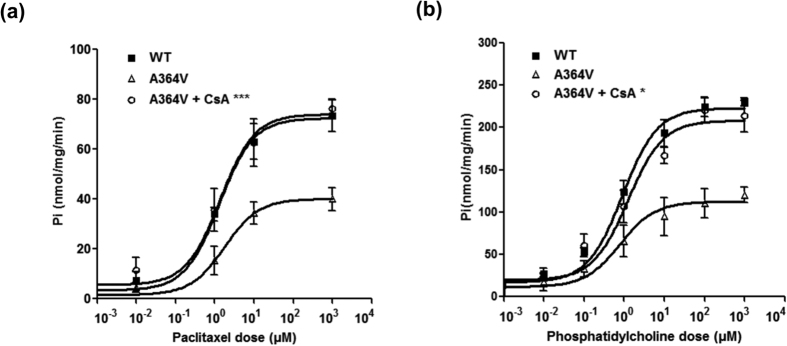
Effect of CsA on the transport activity of the mutant A364V. Membrane vesicular ATPase assays were performed using paclitaxel (**a**) or phosphatidylcholine (**b**) after treatment with CsA. Data shown represent mean ± SD from three independent experiments, and the *P* values for comparison between the results obtained before and after CsA treatment, were calculated using Student’s *t* test. ^*^P < 0.05, ^***^P < 0.001 vs. transport activity of A364V without CsA treatment.

A previous study by Gautherot *et al.*^9^ showed that a trafficking-defective mutation of *ABCB4* was functionally rescued by cyclosporin A. In their study, cyclosporin A produced a dose-dependent increase in MDR3 expression. Therefore, we tested whether a trafficking-defective mutant of *ABCB4*, A364V could be rescued by cyclosporin A. To examine it, we performed cell surface biotinylation after cells were treated with 10 μM cyclosporine A. As a result, we observed that cyclosporin A significantly increased MDR3 expression levels of A364V (Fig. 3). Our finding was similar to those of a previous study^9^. Then, to examine the effect of cyclosporin A on the transport activity of A364V, membrane vesicular ATPase assays were performed after treatment with cyclosporin A. We found that cyclosporin A increased the transport activity of this mutant significantly in the ATPase assays using paclitaxel or phosphatidylcholine (Fig. 5).

## Discussion

Recently, screening of genomic DNA samples from 68 PFIC3 patients identified 29 mutations, 25 of which were novel^7^. In this study, authors examined the effect of each mutation on the protein tertiary structure using a three-dimensional model of MDR3. MDR3 consists of twelve TMs, six intracellular domains (ICDs), six extracellular loops (ECs), and a linker peptide connecting the N-terminal to the C-terminal transmembrane domain-nucleotide binding domain (TMD-NBD). Most mutations were located in the NBDs, TMs, or ICDs, which may be involved in substrate binding and translocation, coupling the energy from ATP hydrolysis to substrate transport, and the conformational change involved in substrate extrusion. Ten mutations were located in TMs, nine in ICDs, and eight in NBDs. However, functional characterization of each mutant was not performed in this previous study.

The present study was conducted to functionally characterize the *ABCB4* mutations reported in the previous study by Degiorgio *et al.*^7^. Among 29 *ABCB4* mutations, we selected 8 missense mutations. Then, we measured the transport activity of each mutant indirectly by ATPase assays using paclitaxel or phosphatidylcholine. we found that three mutants showed significantly decreased transport activities, whereas the other five mutants had similar transport activities compared to that of the wild type. These results are unexpected, however, two mutants, A250P and F357L, were initially identified as in cis with M630V and T775M, respectively^7^, suggesting that F357L or A250P is probably not sufficient to cause loss-of-function of MDR3 transport activity by itself. In addition, the pathogenicity of A286V, V475A and T715I may depend on the trans-associated mutation, as shown for NPHS2 R229Q^26^. In other words, these alleles lead to a disease phenotype only when it is associated specifically with certain *ABCB4* mutations. For three mutants which exhibited decreased transport activities, we found that the reduced expression for A364V, located in the ICDs, might be due to proteasomal and lysosomal degradation. In the case of A737V and A1193T, the results from the cell surface biotinylation, immunoblotting, and immunofluorescence experiments indicated that decreased transport activity of these mutants did not result from the reduced MDR3 expression on the plasma membrane. Recently Gautherot *et al.*^27^ reported that two *ABCB4* mutants, T34M and R47G, resulted in abnormal phosphorylation of its N-terminal domain, leading to decreased secretion of phosphatidylcholine while the targeting to the plasma membrane of those mutants was comparable with the wild type *ABCB4*. Further *in vitro* study would be necessary to elucidate the cause of decreased transport activity of A737V and A1193T.

Most of the ABC transporters are expressed on the plasma membrane of cells, where they transport various substrates. Serious human disease can occur if the transporters are not expressed on the cell surface properly due to folding defects^28^. The correction of folding defects caused by specific mutations, through alteration of intracellular environment or addition of pharmacological compounds, is a valuable approach for the treatment of some genetic diseases. For example, ∆F508 of cystic fibrosis conducting regulator (CFTR), the most common mutation in patients with cystic fibrosis, impairs CFTR folding^29^. Many studies reported that folding and trafficking defects of ∆F508 could be partially rescued by various modulators such as thapsigargin, sodium 4-phenylbutyrate, and aminoarylthiazoles or by overexpression of heat shock protein 70 or Golgi reassembly stacking proteins^9^,^29^,^30^,^31^. In the case of the other ABC transporters, a few approaches have focused on correcting trafficking-defective mutations. For example, a processing mutant of *ABCB1* could also be rescued by substrates or modulators of MDR1^2^,^29^. In particular, it has been shown that the pharmacological chaperone cyclosporin A, a known inhibitor of MDR1, increases the levels of total and matured protein of MDR1 by stabilizing the mutant protein^32^. Recently, two separate studies reported that an *ABCB4* trafficking-defective mutation, I541F, could be rescued by low temperature or addition of cyclosporin A, which could correct the folding defects introduced by this mutation^8^,^9^. Similarly, in this study, we found that the trafficking defect induced by the mutation A364V could be rescued by cyclosporin A. We also found that the transport activity of this mutant was restored with cyclosporin A.

In some PFIC3 patients with mild symptoms, therapy with ursodeoxycholic acid could be effective^5^. However, most PFIC3 patients are ultimately candidates for liver transplantation. Although solid organ transplantation, including liver transplantation, is regarded as one of the most significant advances in medical science, it still faces various challenges such as organ unavailability, postoperative complications, and high costs^33^,^34^. In the case of pediatric liver transplantation, there are additional problems such as growth retardation due to prolonged steroid exposure, lower physical and psychosocial function after liver transplantation, graft loss, and preoperative malnutrition^35^,^36^. The identification of successful mechanism-based drugs for the treatment of PFIC3 is necessary to improve the quality of life in pediatric PFIC3 patients.

The present study has a limitation in that the transport activity or phosphatidylcholine flippase activity was not measured directly. ABC transporters, including MDR3, use ATP as an energy source to transport their substrates^37^; we measured this ATPase activity and thus, indirectly evaluated the transporter activities of wild type and mutant *ABCB4*. However, functional study of transporters using the ATPase assay has been reported previously^38^,^39^. In particular, Kwak *et al.*^39^ investigated the transport activity of MDR1 using two different methods, ATPase assays and direct transport assays using rhodamine 123 and reported that the results from the ATPase assays were comparable with those from the direct assays.

In conclusion, we have characterized a few *ABCB4* mutations found in PFIC3 patients and found that the A364V, A737V, and A1193T mutants show significantly decreased transport activities, as measured indirectly using the ATPase assay. The mechanisms by which these *ABCB4* mutants may decrease transport activities were determined in this study: A364V, a mutant in the ICDs, was trafficking-defective, while others did not affect MDR3 expression on the plasma membrane. We reported that both the expression of MDR3 on the plasma membrane and the transport activity of this trafficking-defective mutant could be rescued by a cyclosporin A. To our knowledge, this is the first study to functionally characterize a large number of *ABCB4* mutations. The functional characterization of *ABCB4* mutations could enable development of mutation-specific treatment regimens for PFIC3 patients in the future.

## Methods

### Construction of *ABCB4* plasmids

To construct a plasmid containing a wild type *ABCB4* gene, the BC_042531 vector was purchased (Thermo Fisher Scientific Inc., Waltham, MA, USA) and subcloned into the pcDNA3.1(+) vector. Mutant-bearing plasmids were produced using QuikChange^®^ II Site-Directed Mutagenesis Kit (Agilent Technologies, Santa Clara, CA, USA). Nucleotide location numbers were assigned according to the *ABCB4* mRNA sequence (GenBank accession number: NM_018849.2). Supplementary Table S1 lists primers used in this study.

### Membrane vesicle preparation

Membrane vesicles were prepared according to a previously described method^40^. *ABCB4* wild type or mutant-bearing plasmids were transfected into human embryonic kidney-293T (HEK-293T) cells using the Calcium Phosphate Transfection Kit (Life Technologies Corporation, Carlsbad, CA, USA). Forty-eight hours later, cells were harvested and subjected to nitrogen cavitation at 350 pounds per square inch, for 15 min. After transfer into a tube containing 0.5 M EDTA, centrifugation was performed twice at 1,900 × *g*, for 10 min each time, to obtain the supernatant. The membrane vesicle fractions were collected by sucrose density gradient centrifugation at 1,000,000 × *g* for 90 min and suspended in buffer containing 250 mM sucrose and 50 mM Tris.

### ATPase assay

The vanadate-sensitive ATPase activity was measured using the SensoLyte^®^ MG Phosphate Assay Kit (AnaSpec, Fremont, CA, USA) for paclitaxel or phosphatidylcholine transport, according to a previously described method^39^. First, membrane vesicles (4 μg/well) were prepared in 4 mM MgCl_2_, 5 mM 3-(N-morpholino)-propanesulfonic acid-Tris (pH 7.0), 4 mM ATP, and various concentrations of paclitaxel or phosphatidylcholine. For the inhibition assay, 150 or 400 μM verapamil was added to each well. Finally, absorbance was measured at 620 nm, using a microplate reader. ATPase activities were determined as the inorganic phosphate liberation, normalized by subtracting the activities of the mock vesicles, without the transfection of *ABCB4* wild type or mutant-bearing plasmids.

### Immunoblotting

The *ABCB4* wild type or mutant-bearing plasmids were transfected into HEK-293T cells by using the Lipofectamine LTX and Plus reagents (Life Technologies). To examine the effect of MG132 or bafilomycin A_1_ on *ABCB4* mutants, cells were treated with 10 μM MG132 or 0.1 mg/ml bafilomycin A_1_ 24 h after transfection. To investigate the effect of cyclosporin A, cells were treated with 10 μM cyclosporin A, 6 h after transfection. Thirty or forty-eight hours after transfection, immunoblotting was performed using the primary antibodies mouse anti-MDR3 antibody (Abcam, Cambridge, UK) or goat anti-β-actin antibody (Santa Cruz Biotechnology, Santa Cruz, CA, USA). The intensity of each band was measured using ImageJ (National Institutes of Health, Bethesda, MD, USA).

### Surface biotinylation assay

Biotinylation experiments were conducted using a Cell Surface Protein Isolation Kit (Thermo Fisher Scientific Inc.), performed according to the manufacturer’s protocol, using the cells obtained from transfection of the *ABCB4* wild type or mutant-bearing plasmids into HEK-293T cells. To examine the effect of cyclosporin A on *ABCB4* mutants, cells were treated with 10 μM cyclosporin A, 6 h after transfection. A rabbit polyclonal anti-Na^+^/K^+^ ATPase α-1 (EMD Millipore, Billerica, MA, USA) and a goat polyclonal anti-Aldolase A (Santa Cruz Biotechnology) antibodies were used as internal standards.

### Immunofluorescence

For immunofluorescence, HEK-293T cells were grown on cover glasses, in a 24-well plate, and the *ABCB4* wild type or mutant-bearing plasmids were transfected into HEK-293T cells. Twenty-four hours after transfection, cells were fixed and permeabilized with cold methanol for 10 min at −20 °C, following which blocking was performed. To detect MDR3, cells were incubated overnight at 4 °C with the anti-MDR3 and anti-BiP (Abcam) or anti-gagantin (Abcam) antibodies. After washing with PBS, Alexa Fluor^®^ 488 rabbit anti-mouse IgG and Alexa Fluor^®^ 594 goat anti-rabbit IgG (Life Technologies) were used as fluorophore-tagged secondary antibodies, and DAPI was used for nucleic acid staining. Confocal images were captured using a confocal laser scanning microscope, and the digital images were analyzed using an LSM Image Examiner (Carl Zeiss, Oberkochen, Germany).

### Statistical analysis

Statistical analysis was performed using the GraphPad Prism 4.0 software package (GraphPad Software Inc., San Diego, CA, USA). The data shown in the ATPase assay, immunoblotting, and surface biotinylation assay represent mean ± SD from more than three separate experiments. *P* values for comparison between the results obtained before and after cyclosporin A treatment were calculated using Student’s *t* test. The other *P* values were calculated using one-way analysis of variance followed by Dunnett’s two-tailed test, and *P* values of less than 0.05 were considered statistically significant.

## Additional Information

**How to cite this article**: Park, H. J. *et al.* Functional characterization of *ABCB4* mutations found in progressive familial intrahepatic cholestasis type 3. *Sci. Rep.* **6**, 26872; doi: 10.1038/srep26872 (2016).

## Supplementary Material

Supplementary Information

## Figures and Tables

**Table 1:** *ABCB4* mutations examined in this study.

cDNA position^a^	Amino acid substitution^b^	Predicted domain	Mutation Taster^c^	PolyPhen2^d^	SIFT^e^	PROVEAN^f^	Frequencies in the dbSNP database^g^	Frequencies in the ExAC database^h^	Frequencies in the EVS database^i^
c.749G > C	p.A250P	ICD2	DC	0.959	Dam	Del	No	No	No
c.857C > T	p.A286V	ICD2	DC	0.990	Dam	Del	No	2/121360 (no homozygote)	No
c.1069T > C	p.F357L	ICD3	DC	0.101	Dam	Del	No	No	No
c.1091C > T	p.A364V	ICD3	DC	0.997	Dam	Del	No	No	No
c.1424T > C	p.V475A	NBD-NH2 ter	DC	0.610	Dam	Del	No	No	No
c.2144C > T	p.T715I	TM2	DC	0.026	Tol	Neu	rs138773456 (MAF = 0.0002)	68/121370 (no homozygote)	AA/AG/GG = 0/10/6493
c.2210C > T	p.A737V	EC4	SNP	0.009	Dam	Neu	rs147134978 (MAF, NA)	3/121404 (no homozygote)	AA/AG/GG = 0/1/6502
c.3577G > A	p.A1193T	NBD-COOH ter	DC	0.966	Dam	Del	No	No	No

Abbreviations are as follows: Dam, damaging; DC, disease causing; Del, deleterious; EC, extracellular loop; ICD, intracellular domain; MAF, minor allele frequency; NA, not available; NBD, nucleotide binding domain; Neu, neutral; No, no data; SNP, single nucleotide polymorphism; ter, terminal end; tol, tolerated; TM, transmembrane domain.

^a^cDNA position is numbered according to human cDNA reference sequence NM_018849.2 (*ABCB4*); +1 corresponds to the A of ATG translation initiation codon.

^b^Amino acid residue is numbered according to human protein reference sequence NP_061337.1.

^c^Mutation taster (http://www.mutationtaster.org/).

^d^PolyPhen-2 prediction score HumVar ranges from 0 to 1.0; 0 = benign, 1.0 = probably damaging (http://genetics.bwh.harvard.edu/pph2/).

^e^Sorting Intolerant From Tolerant (http://sift.jcvi.org/www/SIFT_chr_coords_submit.html).

^f^Protein Variation Effect *A*nalyzer (http://provean.jcvi.org/seq_submit.php).

^g^dbSNP database (http://www.ncbi.nlm.nih.gov/SNP).

^h^Exome Aggregation Consortium browser (http://exac.broadinstitute.org/).

^i^Exome Variant Server (http://evs.gs.washington.edu/EVS/).

**Table 2:** Kinetic values of *ABCB4* wild type or its mutants in the ATPase assay using paclitaxel.

	V_max_ (nmol mg^−1^ per min)	K_m_(μM)	V_max_/K_m_ ratio (nmol mg^−1^ min^−1^ per μM)
WT	72.27 ± 15.60	1.11 ± 0.14	65.75 ± 15.39
A250P	62.51 ± 14.20	1.27 ± 0.15	49.49 ± 10.64
A286V	72.97 ± 17.00	1.03 ± 0.21	72.65 ± 17.20
F357L	53.41 ± 11.71	1.10 ± 0.18	48.06 ± 5.02
A364V	44.29 ± 14.94^*^	1.35 ± 0.22	33.81 ± 13.21^*^^*^
V475A	59.81 ± 15.50	1.20 ± 0.21	49.78 ± 8.95
T715I	58.64 ± 16.33	1.13 ± 0.21	51.90 ± 12.48
A737V	38.63 ± 8.54^*^^*^	1.12 ± 0.15	34.88 ± 9.74^*^^*^
A1193T	46.58 ± 5.47^*^	1.18 ± 0.19	40.05 ± 6.39^*^

Data (mean ± SD) were from 5 separate experiments.

^*^P < 0.05, ^*^^*^P < 0.01; multiple comparisons were performed using one-way analysis of variance followed by Dunnett’s two-tailed test. The values of wild type were used as control.
