# Supplementary material for: Functional characterization of ABCB4 mutations found in progressive familial intrahepatic cholestasis type 3
Source: Sci Rep. 2016 Jun 3;6:26872. doi: 10.1038/srep26872 (PMC4891722; doi:10.1038/srep26872)

Supplementary Information

For

Functional characterization of *ABCB4* mutations found in progressive familial intrahepatic cholestasis type 3

Hyo Jin Park[1,4], Tae Hee Kim[1,4], So Won Kim[2], Shin Hye Noh[3], Kyeong Jee Cho[3], Choe Choi[3], Eun Young Kwon[1], Yang Ji Choi[1], Heon Yung Gee[3,*], Ji Ha Choi[1,*]

[1]Department of Pharmacology, Tissue Injury Defense Research Center, School of Medicine, Ewha Womans University, Seoul, Korea

[2]Department of Pharmacology and the Institute for Clinical and Translational Research, Catholic Kwandong University College of Medicine, Gangneung, Korea

[3]Department of Pharmacology, Brain Korea 21 PLUS Project for Medical Sciences, Yonsei University College of Medicine, Seoul, Korea

[4]H.J.Park and T.H.Kim contributed equally to this study.

[*]corresponding authors: jihachoi@ewha.ac.kr or hygee@yuhs.ac

***Correspondence to**

Ji Ha Choi, M.D., Ph.D.

Department of Pharmacology, Tissue Injury Defense Research Center

School of Medicine, Ewha Womans University, Seoul, Korea

1071 Anyangcheon-ro, Yangcheon-gu, Seoul 07985, Korea

Phone: +82-2-2650-5746; Fax: +82-2-2653-8891; E-mail: jihachoi@ewha.ac.kr

Heon Yung Gee, M.D., Ph.D.

Department of Pharmacology, Yonsei University College of Medicine

Yonsei-ro 50-1, Seodaemun-gu, Seoul 03722, Korea

Phone: +82-2-2228-0755; Fax: +82-2-313-1894; E-mail: hygee@yuhs.ac

Supplementary Table S1. Oligonucleotide primers used in the construction of *ABCB4* plasmids

Primes for *ABCB4* cloning[a]	
Sense (HindIII site)	5'-CCC **AAG CTT** ACC ATG GAT CTT GAG GCG GCA-3'
Antisense (XbaI site)	5’-GC**T CTA GA**G CAA AAG TTC ATA AGT TCT GTG TCC-3’
Primers for *ABCB4* mutagenesis PCR[b]	
A250P	5'-GAA CTA GCT GCT TAT **<u>C</u>**CA AAA GCA GGC GCC G-3’
A286V	5'-GCT GGA AAG GTA TCA GAA ACA TTT AGA AAA TG**<u>T</u>** CAA AGA GAT TGG AAT-3’
F357L	5'-CAG GAT GCC CCA TGT ATT GAT GCT **<u>C</u>**TT GCC AAT GCA AGA GGA GCA-3‘
A364V	5'-GAT GCT TTT GCC AAT GCA AGA GGA GCA G**<u>T</u>**A TAT GTG ATC TTT GAT AT-3‘
V475A	5'-GAG GGA AAT CAT TGG TGT GG**<u>C</u>** GAG TCA GGA GCC GGT GCT GTT-3‘
T715I	5’-GGC CCT ACT TTG TCG TGG GAA **<u>T</u>**AG TAT GTG CCA TTG CCA ATG G-3’
A737V	5’-CAG TCA TAT TCT CAG AGA TCA TAG **<u>T</u>**GA TTT TTG GAC CAG GCG ATG A-3’
A1193T	5’-GGT CAA AAA CAG AGG ATT GCT ATT **<u>A</u>**CC CGA GCC CTC ATC AGA CAA-3’

[a]The restriction endonuclease sites were marked by bold-faced letters.
[b]The SNP sites were marked by bold-faced letters with underlines.

Supplementary Figure S1. Effect of verapamil on transport activity. Transport activity for paclitaxel or phosphatidylcholine of the MDR3 wild type was measured after treatment with verapamil.

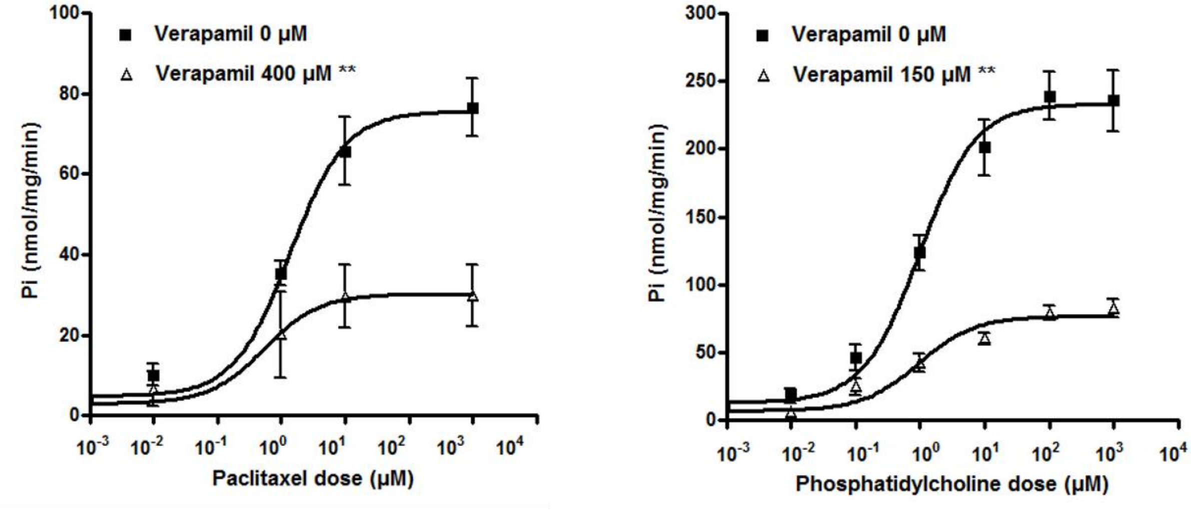

Supplementary Figure S2. Effect of *ABCB4* mutants on MDR3 expression. MDR3 expression was investigated after transfection with *ABCB4* mutant plasmids, which showed similar transport activities, compared to that of the wild type (WT). EV, empty vector (pcDNA3.1(+))

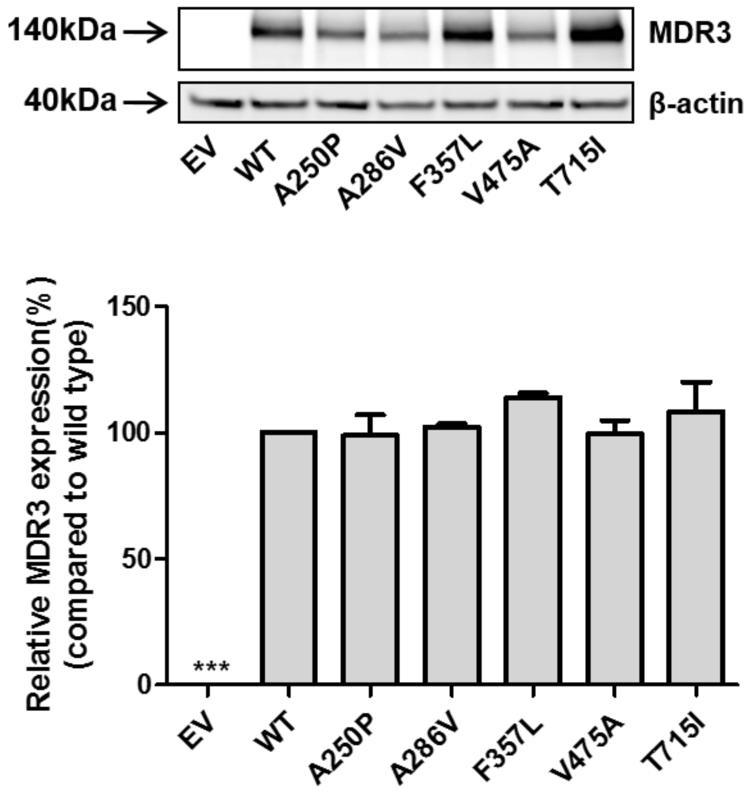

Supplement: Supplementary Information [file srep26872-s1.pdf]
